# Antitumor effect and toxicity of free rhodium (II) citrate and rhodium (II) citrate-loaded maghemite nanoparticles in mice bearing breast cancer

**DOI:** 10.1186/1477-3155-11-4

**Published:** 2013-02-16

**Authors:** Marcella Lemos Brettas Carneiro, Raphael CA Peixoto, Graziela A Joanitti, Ricardo GS Oliveira, Luis AM Telles, Ana L Miranda-Vilela, Anamélia L Bocca, Leonora MS Vianna, Izabel CR da Silva, Aparecido R de Souza, Zulmira GM Lacava, Sônia N Báo

**Affiliations:** 1 Departamento de Biologia Celular, Instituto de Ciências Biológicas, Universidade de Brasília (UnB), 70.910-900, Brasília-DF, Brazil; 2 Departamento de Patologia, Faculdade de Medicina, Universidade de Brasília, 70.919-970, Brasília-DF, Brazil; 3 Faculdade LS, 72.020-111, Brasília-DF, Brazil; 4 Instituto de Química, Universidade Federal de Goiás, Campus Samambaia, 74.001-970, Goiânia, Brazil

## Abstract

**Background:**

Magnetic fluids containing superparamagnetic iron oxide nanoparticles represent an attractive platform as nanocarriers in chemotherapy. Recently, we developed a formulation of maghemite nanoparticles coated with rhodium (II) citrate, which resulted in *in vitro* cytotoxicity enhanced up to 4.6 times when compared to free rhodium (II) citrate formulation on breast carcinoma cells. In this work, we evaluate the antitumor activity and toxicity induced by these formulations in Balb/c mice bearing orthotopic 4T1 breast carcinoma.

**Methods:**

Mice were evaluated with regard to the treatments’ toxicity through analyses of hemogram, serum levels of alanine aminotransferase, iron, and creatinine; DNA fragmentation and cell cycle of bone marrow cells; and liver, kidney and lung histology. In addition, the antitumor activity of rhodium (II) citrate and maghemite nanoparticles coated with rhodium (II) citrate was verified by tumor volume reduction, histology and immunohistochemistry.

**Results:**

Regarding the treatments’ toxicity, no experimental groups had alterations in levels of serum ALT or creatinine, and this suggestion was corroborated by the histopathologic examination of liver and kidney of mice. Moreover, DNA fragmentation frequency of bone marrow cells was lower than 15% in all experimental groups. On the other hand, the complexes rhodium (II) citrate-functionalized maghemite and free rhodium (II) citrate led to a marked growth inhibition of tumor and decrease in CD31 and Ki-67 staining.

**Conclusions:**

In summary, we demonstrated that both rhodium (II) citrate and maghemite nanoparticles coated with rhodium (II) citrate formulations exhibited antitumor effects against 4T1 metastatic breast cancer cell line following intratumoral administration. This antitumor effect was followed by inhibition of both cell proliferation and microvascularization and by tumor tissue injury characterized as necrosis and fibrosis. Remarkably, this is the first published report demonstrating the therapeutic efficacy of maghemite nanoparticles coated with rhodium (II) citrate. This treatment prolonged the survival period of treated mice without inducing apparent systemic toxicity, which strengthens its use for future breast cancer therapeutic applications.

## Background

Breast cancer represents the main cause of death in women worldwide due to the high metastatic capacity of this disease [1]. Nowadays, chemotherapy is the most commonly used treatment in the therapeutic approach; however, conventional chemotherapy has shown low efficacy when the disease is not treated in early stages [2]. As a matter of fact, chemotherapy drugs are not specific and act both on tumor and normal cells, causing side effects [3]. Thus, the development of therapeutic strategies such as drug delivery systems (DDS) represents an area of great interest in cancer research [4-6].

Through the development of nanotechnology, nanomaterials designed to work as DDS are increasingly being used in nanomedicine. This field has advanced, presenting innovative approaches, allowing the improvement of cancer therapeutic treatments [7]. Among various materials used for DDS, magnetic fluids containing superparamagnetic iron oxide nanoparticles (SPIOs) represent an attractive platform as nanocarriers in chemotherapy [8]. SPIOs ranging from 10 to 500 nm in size can accumulate inside the interstitial space in tumors, since the blood vessel wall becomes more permeable than in the normal tissue state. This effect, added to the poor lymphatic drainage in tumor and the tumor microvasculature, which has a discontinuous and loose nature [9,10], is known as enhanced permeability and retention (EPR) and is currently considered an effective way to bring drugs into tumors, especially drug-loaded nanocarriers [11]. Thus, SPIOs have shown promising results in the field of oncology, acting as an efficient DDS for controlled drug release and penetration in solid tumors [4,7,12].

In the last decade, an increasing number of investigations using several types of iron oxide-based nanoparticles have been carried out [13]. However, only maghemite (γ-Fe_2_O_3_) and magnetite (Fe_3_O_4_) are able to fulfill the necessary requirements for biomedical applications, due to their superior biocompatibility with respect to other magnetic materials [14]. Nowadays, magnetic nanoparticles are being associated with several chemotherapy drugs, such as doxorubicin [15], docetaxel [16], methotrexate [17], tamoxifen [18], paclitaxel and cisplatin [19].

**Figure 1:**
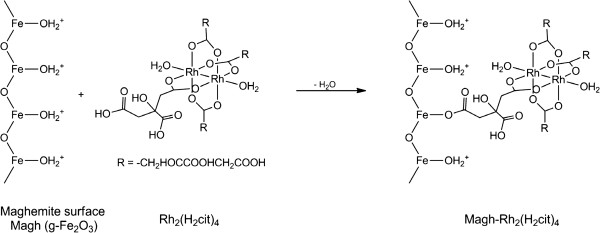
Representation of rhodium (II) citrate showing chemical bond between rhodium (II) citrate and maghemite nanoparticles.

Rhodium carboxylates, a class of metal complexes, have shown promising antitumor activity in cisplatin-resistant cell lines [20]; they present significant cytostatic activity in tumors L1210, Ehrlich ascites carcinoma, sarcoma 180 and P388 and melanoma B16 [21]. Among the class of rhodium carboxylates, rhodium (II) citrate showed cytotoxic activity, cytotoxic and antitumor Ehrlich breast carcinoma [22]. Interestingly, the citrate ligand of this complex has the ability to functionalize SPIOs to promote stability and biocompatible stable colloidal suspensions, making them suitable for applications in drug delivery systems. Whereas the maghemite nanoparticle surface is easily functionalized by ions or molecules, the free carboxyl groups, present in the chemical structure of rhodium (II) citrate, can bind to Fe-OH surface of nanoparticles, a reaction that occurs with the elimination of water and formation of the Fe-OCO chemical bond (Figure 1).

Recently, our group developed a formulation of maghemite nanoparticles (NPs) coated with citrate (Magh-cit) and rhodium (II) citrate (Magh-Rh_2_(H_2_cit)_4_), and we have shown that Magh-Rh_2_(H_2_cit)_4_ enhanced cytotoxicity on breast carcinoma cells by up to 4.6 times when compared to free rhodium (II) citrate (Rh_2_(H_2_cit)_4_). Likewise, we verified that Magh-Rh_2_(H_2_cit)_4_ induced higher *in vitro* cytotoxicity on breast carcinoma cells than on breast normal cells [23]. Thus, in the present study, we aimed to evaluate the antitumor activity and systemic toxicity induced by intratumoral injections of Rh_2_(H_2_cit)_4_ and Magh-Rh_2_(H_2_cit)_4_ in Balb/c mice bearing 4T1 breast carcinoma.

## Results and discussion

### Systemic toxicity assessment in mice bearing 4T1 breast carcinoma

No skin ulceration, weight loss or evident alterations were found, indicating the good tolerance of mice to the treatments. There were also no significant differences in the analyzed hematological parameters when Rh_2_(H_2_cit)_4_ and Magh-Rh_2_(H_2_cit)_4_ groups were compared with the control (Table 1). The evaluation of blood cells is an integral part of the routine assessment of healthy and diseased animals [24]. Moreover, combining hematology analysis with biochemical serum dosages, we have an important tool for pathologic analysis.

**Table 1:** **Effects of Rh**_**2**_**(H**_**2**_**cit)**_**4,**_**Magh-Rh**_**2**_**(H**_**2**_**cit)**_**4**_**and Magh-citrate on hematology and biochemical parameters of female Balb/c mice 23 days after animals’ tumor transplantation**^**1**^

**Treatment**	**Control**	**Rh**_**2**_**(H**_**2**_**cit)**_**4**_	**Magh-Rh**_**2**_**(H**_**2**_**cit)**_**4**_	**Magh-citrate**
**Hematology parameters**				
**WBC (/mm3)**	18445.50± 6020.68	24555.00 ± 9693.69	45774.00 ± 11995.51	59060.00 ± 25390.07
**RBC (x109/mm3)**	8.69±0.27^B^	10.82± 1.19	10.07 ± 1.03	10.17 ± 0.86
**HGB (g/dL)**	13.60±0.42	14.75 ± 0.33	14.26 ± 0.14	13.36 ± 0.85
**HCT (%)**	41.60±1.38	44.62 ± 0.96	44.40 ± 0.39	41.09 ± 2.51
**Lymphocytes (%)**	28.75±6.65	38.50 ± 13.88	10.80± 3.35	18.71 ± 7.66
**Rods (%)**	1.25±1.25	0.50 ± 0.50	0.00 ± 0.00	1.43 ± 0.65
**Segmented(%)**	67.00±7.14	57.67 ± 13.40	84.00 ± 3.36	70.57 ± 7.40
**Eosinophils (%)**	0.00±0.00	0.00 ± 0.00	0.00 ± 0.00	0.14 ± 0.14
**Basophils (%)**	0.25±0.25	0.17 ± 0.17	0.00 ± 0.00	0.14 ± 0.14
**Monocytes (%)**	2.75±0.75	3.17 ± 1.01	5.20 ± 1.11	9.00 ± 4.94
**Biochemical parameters**	25.20±6.22	25.50 ± 3.57	20.86 ± 2.15	21.33 ± 3.38
**ALT (U/L)**	0.10±0.00	0.10 ± 0.00	0.10 ± 0.00	0.13 ± 0.02
**Creatinine (mg/dL)**	125.60±18.59	143.67 ± 14.22	127.57 ± 12.10	112.33 ± 15.23
**Serum Fe (mg/dL)**				

Legend: WBC (White Blood Cells); RBC (Red Blood Cells); HGB (Hemoglobin); HCT (Hematocrit); ALT (alanine transaminase).

^1^Values are represented as mean ± standard error.

Note: all treatments have no statistical difference when compared with untreated control mice (P>0.05).b.

**Figure 2:**
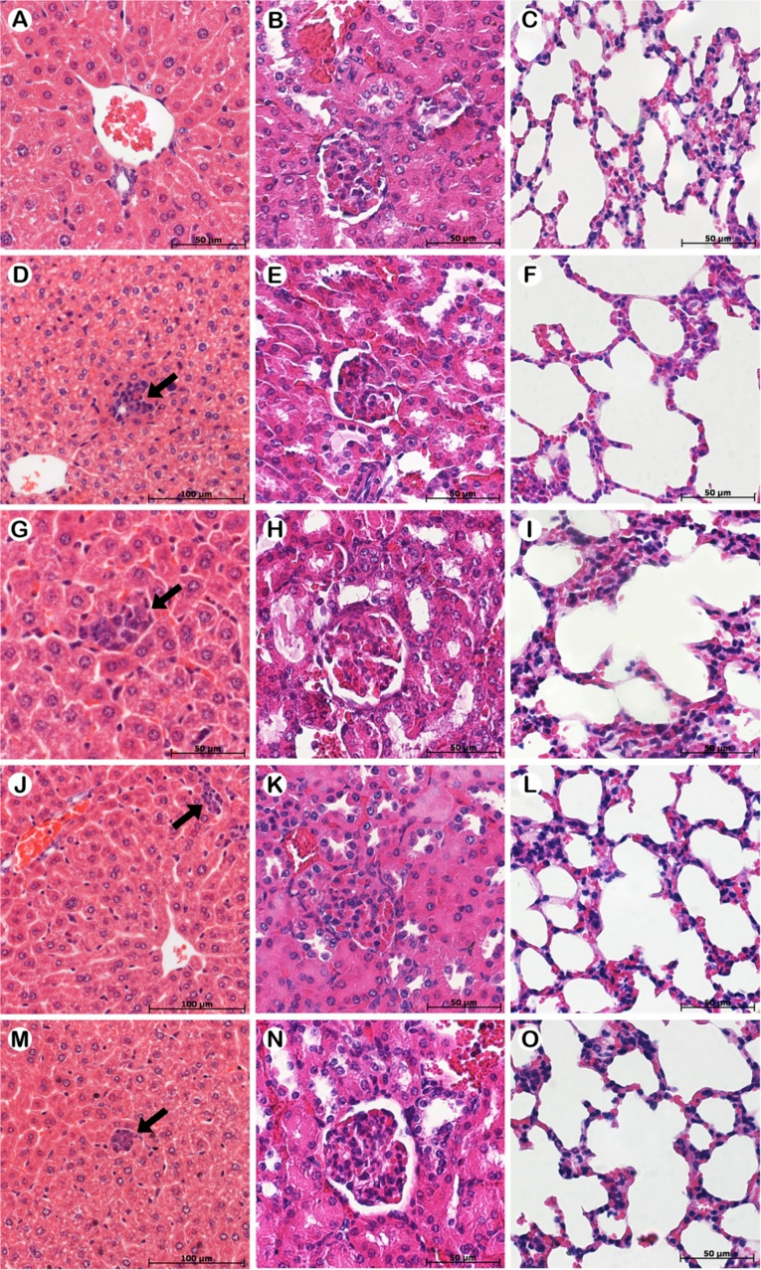
**Representative histopathology of liver, kidney and lung from Balb/c mice healthy and Balb/c mice bearing 4T1 breast carcinoma in different treatment groups.** The animals were treated with Rh_2_(H_2_cit)_4_ and Magh-Rh_2_(H_2_cit)_4_ (1050 μM total dose equimolar of Rh_2_(H_2_cit)_4_), and they were sacrificed on the 23^rd^ day after tumor inoculation. Tumor sections were stained with H&E. **A-C**) Healthy mice and mice bearing 4T1 breast carcinoma without treatment (**D-F**) or treated with Rh_2_(H_2_cit)_4_ (**G-I**), Magh-Rh_2_(H_2_cit)_4_ (**J-L**) and Magh-citrate (**M-O**).

No experimental groups had alterations in levels of serum ALT or creatinine. This suggestion was corroborated by the histopathologic examination of liver and kidney of treated-mice (Figure 2), which showed no morphological changes, although a focal inflammatory infiltrate in the liver was observed (Figure 2D, 2G, 2J, and 2M) from mice bearing 4T1 breast carcinoma. However, this is a benign reaction, known as an inflammatory pseudotumor (IPT) that has been described in both sexes, at all ages, and in almost any location. IPTs are the expression of diverse inflammatory processes of unknown etiology that may be accompanied by a tumor-like mass and affect several organs of the body. Although the usual location of IPT is the lung, the most common extrapulmonary site of IPT is the liver [25]. Thus, the results indicate that this focal inflammatory infiltrate was a liver response to tumor implantation, since no nanoparticles were observed in the analyzed organs.

**Figure 3:**
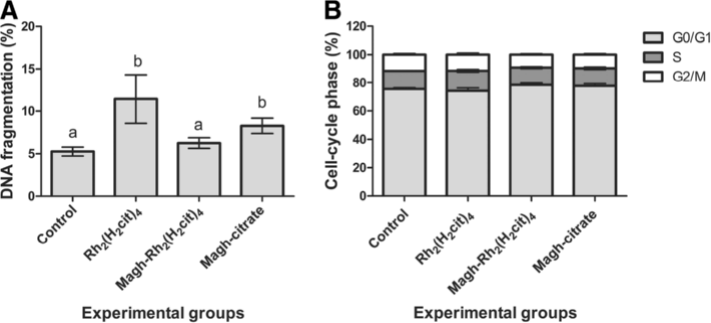
**Toxicity of Rh**_**2**_**(H**_**2**_**cit)**_**4 **_**and Magh-Rh**_**2**_**(H**_**2**_**cit)**_**4**_**on bone marrow cells of mice-bearing 4T1 breast carcinoma.** Mice (n=8) were treated with Rh_2_(H_2_cit)_4_ and Magh-Rh_2_(H_2_cit)_4_ with seven equimolar doses of Rh_2_(H_2_cit)_4_ (1050 μM total dose) until 23^rd^ day. Subsequently, bone marrow cells were stained with propidium iodide and analyzed by flow cytometry. (**A**) DNA fragmentation (**B**) Cell cycle. Data were expressed as percentages as mean ± SEM. Different letters indicate significant difference among experimental groups (p<0.05).

Evaluation of the potential genotoxic effects on normal cells is crucial during the development of new anticancer drugs in order to avoid severe DNA damage to non-targeted cells [26]. DNA fragmentation frequency of BM cells was lower than 15% in all experimental groups, with the Magh-Rh_2_(H_2_cit)_4_ group showing DNA very similar fragmentation frequency to the control group, and Rh_2_(H_2_cit)_4_ and Magh-citrate groups showing slightly higher values (Figure 3A). No significant difference in the BM cell cycle was observed among the groups (Figure 3B). Thus, the results indicate that Magh-Rh_2_(H_2_cit)_4_ treatment did not lead to significant interference in DNA integrity or cell division of BM cells in mice. The present data are corroborated by previous reports showing that no genotoxic effects were observed in mice treated with nanoparticle formulations containing maghemite, cobalt ferrite or magnetite [27-29].

### Tumor volume regression and survival test

**Figure 4:**
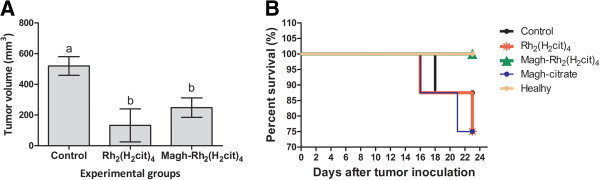
**Antitumor effect and survival curve.** The mice were treated with Rh_2_(H_2_cit)_4_ or Magh-Rh_2_(H_2_cit)_4_ and received seven equimolar doses of Rh_2_(H_2_cit)_4_ (1050 μM total dose) until 23^rd^ day after tumor inoculation. **A**) Antitumor effects of Rh_2_(H_2_cit)_4_ and Magh-Rh_2_(H_2_cit)_4_. **B**) Survival curve for tumor-bearing mice. Values represent mean values ± standard error (n=8/each group) and different letters indicate statistical difference among treatments (p<0.05).

The complexes rhodium (II) citrate-functionalized maghemite (Magh-Rh_2_(H_2_cit)_4_) and free rhodium (II) citrate (Rh_2_(H_2_cit)_4_) led to a marked growth inhibition of tumor, when compared to the control group (p<0.05). These treatments induced a significant tumor volume reduction of about 75% and 52%, respectively, which demonstrated efficient antitumor activity of these complexes on the 4T1 metastatic breast cancer cell line (Figure 4A). Antitumor effects were not observed in mice treated with citrate-functionalized-maghemite (data not shown).

Citrate-functionalized-maghemite has been attested as providing successful nanoparticles in the production of biocompatible and stable magnetic fluids [30,31]; however, our work was the first to investigate and demonstrate the antitumor effect of rhodium (II) citrate associated with nanoparticles on *in vivo* 4T1 orthotopic breast carcinoma model. Previous studies, conducted by Zyngier *et al* (1989), demonstrated that free rhodium (II) citrate has significant antitumor, cytotoxic, and cytostatic activity in Swiss mice bearing ectopic breast carcinoma model (Ehrlich cell line) [22], which is in agreement with our results.

Nevertheless, two important aspects should be taken into consideration. First, unlike the study of Zyngier *et al* (1989), where the process of tumor implantation was ectopic, we transplanted tumor cells in the mammary gland (orthotopic implantation), which is the original environment of this cell line. This represents an important factor when developing therapies that are able to target not only the tumor but also its microenvironment components [32]. In view of this, our results are of great importance for the study of the effects of anticancer drugs in the mammary microenvironment. Second, our study is the first to evaluate *in vivo* the effects of the association of rhodium (II) citrate to nanoparticles. We suppose that these associations could provide higher therapeutic efficacy than free formulations, since drug-loaded nanocarriers potentially provide controlled time release of the drug beyond modification of drug pharmacokinetics and biological distribution [33]. Moreover, the interaction of nanoparticles with the biological environment is very important for designing strategies that should be selective at the pharmacological site, especially in the breast, where the blood flow and lymphatic drainage is intense [34].

Furthermore, we also evaluated the antitumor effect of Rh_2_(H_2_cit)_4_ and Magh-Rh_2_(H_2_cit)_4_ by the survival analysis of mouse groups, during the experimental period of 23 days. Deaths in the mouse control group (with untreated tumors) started on experimental day 16 after tumor implantation. On the 18^th^ and 21^st^ days, two deaths occurred in each group of animals treated with Rh_2_(H_2_cit)_4_ or Magh-citrate, and one death in the control group. All mice in the Magh-Rh_2_(H_2_cit)_4_ group survived until the end of the experimental period (23 days) (Figure 4B). Magh-Rh_2_(H_2_cit)_4_ complex exhibited antitumor effect against 4T1 metastatic mouse breast carcinoma, without apparent systemic toxicity and a significant increase in treated mice survival period (period of 23 days). Zyngier and Kimura (1989) showed a percentage of 78.8% survival in Swiss mice treated with Rh_2_(H_2_cit)_4_ after implantation of Ehrlich tumor cells, in a period ranging from 23 to 27 days [22]. These findings are consistent with the present study since we observed the same survival percentages in Balb/c mice treated with free Rh_2_(H_2_cit)_4_ (about 75%), after 4T1 cell implantation for a similar period of time (Figure 4B).

In our previous study, we reported that Rh_2_(H_2_cit)_4_ induced cytotoxicity on both breast cancer and breast normal cells, *in vitro*. However, rhodium associated with the nanoparticles (Magh-Rh_2_(H_2_cit)_4_) was more cytotoxic to breast cancer cell lines [23]. These findings are consistent with the present *in vivo* study since mice treated with Magh-Rh_2_(H_2_cit)_4_ showed a decrease in tumor volume, without occurrence of deaths or macro and microscopic alterations.

Although rhodium (II) citrate-coated maghemite nanoparticles have never been described before, another study reported the association of rhodium (II) citrate with hydroxypropyl-beta-cyclodextrin, macrocyclic oligosugars from biodegradable polymer. In this study, the association with these oligosugars led to the minimization of nonspecific toxicity since they increased the efficiency of encapsulation and the duration of rhodium (II) citrate release [35].

On the other hand, some strategies have been used to increase the specificity of antitumor agents by associating drugs with nanocarriers. SPIOs, for instance, are considered one of the most important nanocarriers due to their inherently low particle size, high magnetization values and ability to load anticancer agents, allowing enhanced therapeutic selectivity provided by local hyperthermia, magnetic targeting and magnetic resonance imaging [36,37]. Furthermore, SPIOs are small enough to escape renal clearance and opsonization processes, thus being able to accumulate easily in the tumor [38]. The Magh-Rh_2_(H_2_cit)_4_ complex used in this study contains nanoparticles with sizes of 10 nm [23] thus being small enough to escape renal clearance and opsonization processes, which can promote a preferential accumulation in the tumor due to its abnormal vascular nature [38]. Hence, the nano-sized property was exploited to take advantage of alterations that occur in tumor cells and adjacent tissues [39].

### Tumor histopathology

Histological analyses showed that the 4T1 tumor model without treatment was invasive and characterized by typical and atypical mitoses, poor differentiation, moderate pleomorphism with neovascularization areas and infiltration of inflammatory cells in tissue (data not shown). Previous reports showed that 4T1 tumor cells display a multistage tumor development pattern with atypical hyperplasia [40], corroborating our findings. The 4T1 orthopic breast tumor is a model that shares similarities with metastatic human breast cancer, thus representing a good model to evaluate the efficacy of anticancer drugs [41].

**Figure 5:**
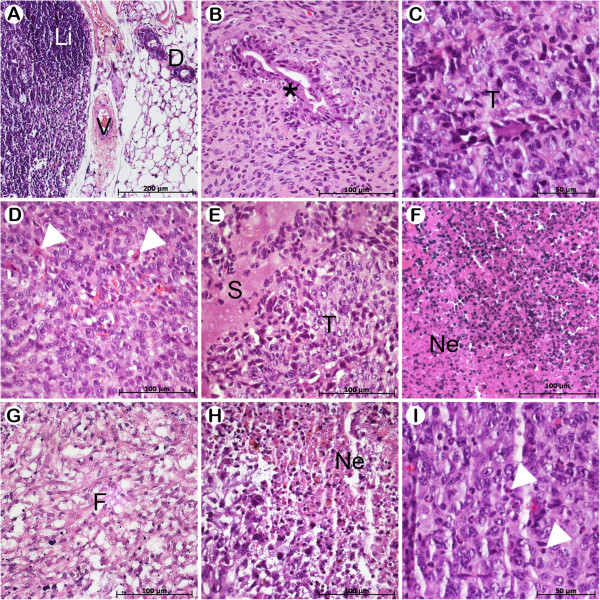
**Representative histopathology of mammary gland and tumors developed from Balb/c mice bearing 4T1 breast carcinoma in different treatment groups.** The animals were treated as described in section 2.3 and tumor slides were stained with H&E. **A**) Healthy group. **B**) Control. **C**) Magh-citrate. **D-F**) Rh_2_(H_2_cit)_4_. **G-I**) Magh-Rh_2_(H_2_cit)_4_. Asterisk indicates changes in the glandular epithelium tumor. Duct (**D**). Fibrosis (**F**). Lymph node (Li). Necrosis areas (N). Secretion (S). Tumor (T). Vessel (V). Apoptosis cells (arrows).

Histopathological characteristics of the different treatment groups are shown in Figure 5. Tumors from control groups (control and Magh-citrate) presented peculiar pleomorphism and were characterized as poorly differentiated. Moreover, in mice treated with Rh_2_(H_2_cit)_4_ we observed inflammatory edema (Figure 5E), as well as the presence of nuclear fragments observed in both Rh_2_(H_2_cit)_4_ and Magh-Rh_2_(H_2_cit)_4_ groups, which may be related to tumor cell death by apoptosis (Figure 5D, I). These results corroborate the tumor regression observed in mice treated with these compositions.

Regarding Rh_2_(H_2_cit)_4_ and Magh-Rh_2_(H_2_cit)_4_ treatments, we observed intense necrosis and fibrosis, demonstrating that its antitumor effectiveness can be associated with cell death triggered in tumor tissue (Figure 5F-H). Moreover, in mice treated with Rh_2_(H_2_cit)_4_ we observed inflammatory edema (Figure 5E), as well as the presence of nuclear fragments observed in Rh_2_(H_2_cit)_4_ and Magh-Rh_2_(H_2_cit)_4_ groups, which may be related to tumor cell death by apoptosis (Figure 5D, I). These results corroborate the tumor regression observed in mice treated with these compositions.

**Figure 6:**
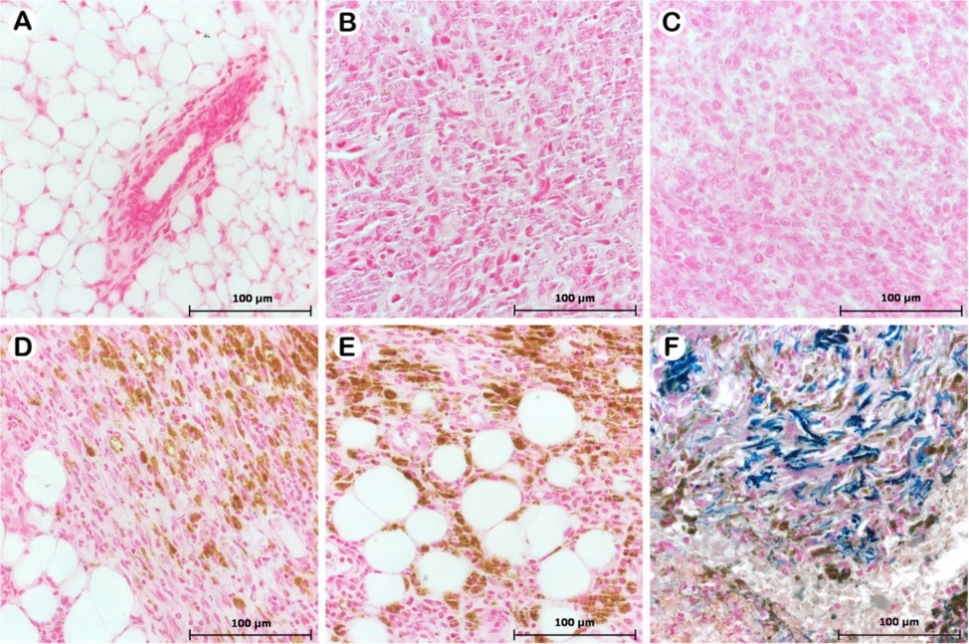
**Histopathology of mammary gland and tumors developed from Balb/c mice bearing 4T1 breast carcinoma in different treatment groups.** The animals were treated as described in section 2.3 and tumor slides were stained with Perls coloration. **A**) Breast tissue of healthy group. **B**) 4T1 tumor cells without treatment (control), with treatment of Rh_2_(H_2_cit)_4_ (**C**), Magh-Rh_2_(H_2_cit)_4_ (**D**) and Magh-citrate (**E**). **F**) Positive control staining Perls Prussian blue (human tissue). The blue-green indicates Fe^2+^ (iron is non-oxidized) and brown indicates the presence of Fe^3+^ (oxidized iron). The presence of maghemite nanoparticles (oxidized iron) was observed, represented by brown coloration.

In Figure 6A the control group tissue is shown, where no nanoparticles were found, while in Figure 6F a positive control is shown for Perls staining in human tissue. We demonstrated the presence of nanoparticles (Magh-Rh_2_(H_2_cit)_4_ and Magh-citrate) on tumor tissue, suggesting that these complexes were uptaken by tumor cells (Figure 6B-E). It has been reported that the accumulation of nanoparticles in cancer cells is useful not only for cancer detection and treatment but also essential to the efficacy of drug investigation [42]. Nanoparticle size, surface chemistry and charge have an intense effect on its internalization capability in cells [43]. Previous studies have demonstrated that anionic magnetic nanoparticles are taken up by smooth muscle cells [44], RAW 264.7 mouse macrophages, human cervix adenocarcinoma cell line, HeLa and melanoma cells [45] by intracytoplasmic vesicles [46]. It is of note that this internalization is responsible for the observed biological effects. We suggest that tumor regression in mice treated with Magh-Rh_2_(H_2_cit)_4_ is correlated to the internalization of these complexes by tumor cells, and consequent tumor injury observed in our histopathological analysis.

### CD31 and Ki-67 immunohistochemistry analysis

**Figure 7:**
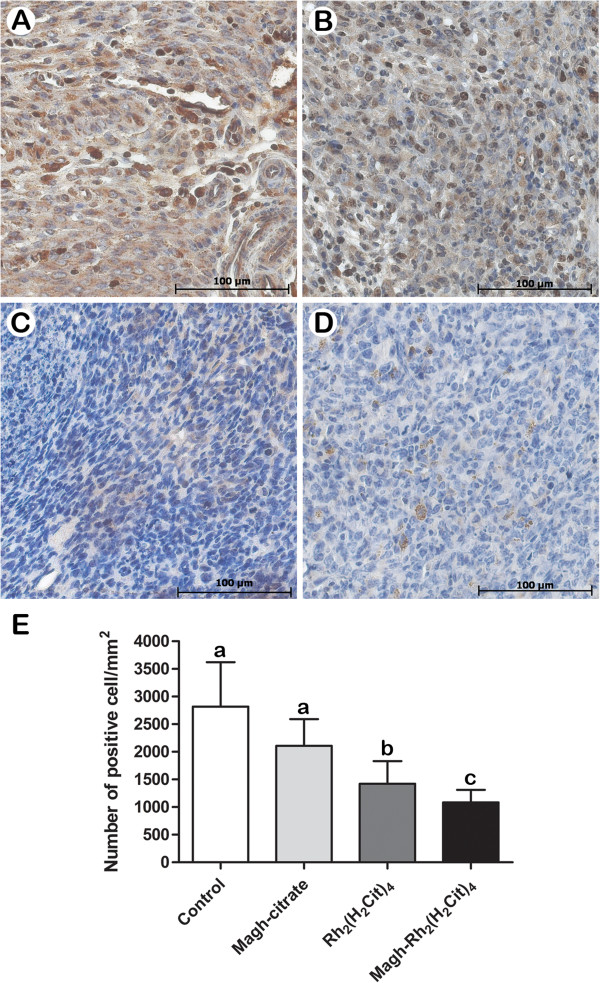
**Inhibition of cell proliferation (Ki67) in 4T1 breast carcinoma tissue.** The mice were treated as described in section 2.3 and tumor slides were immunostained with anti-Ki67. **A**) Control, **B**) Magh-citrate, **C**) Rh_2_(H_2_cit)_4_**D**) Magh-Rh_2_(H_2_cit)_4_. **E**) Quantification of Ki67 staining indicated that treatments with Rh_2_(H_2_cit)_4_ and Magh-Rh_2_(H_2_cit)_4_ inhibited tumor cell proliferation by nearly 50% within solid tumor sections when compared to untreated control mice. Data represent mean values ± standard error (n=4/each group) and different letters indicate statistical difference among treatments (p<0.05).

**Figure 8:**
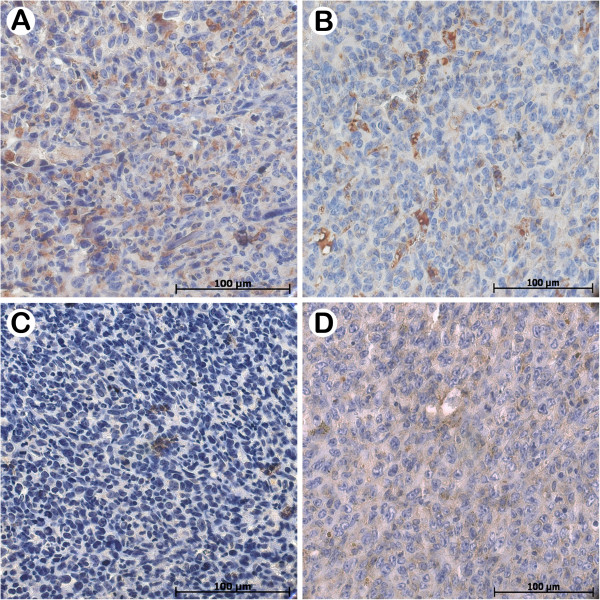
**Effect of Rh**_**2**_**(H**_**2**_**cit)**_**4 **_**and Magh-Rh**_**2**_**(H**_**2**_**cit)**_**4**_**in inhibition of CD31 on mammary gland from Balb/c mice bearing 4T1 breast carcinoma in different treatment groups.** The animals were treated as described in section 2.3 and tumor slides were immunostained with anti-CD31. **A**) Control, **B**) Magh-citrate, **C**) Rh_2_(H_2_cit)_4_**D**) Magh-Rh_2_(H_2_cit)_4_.

We observed that administration of Rh_2_(H_2_cit)_4_ and Magh-Rh_2_(H_2_cit)_4_ led to significant decrease of CD31 and Ki-67 staining compared with controls (Figures 7 and 8), corroborating many studies that have associated the reduction of Ki-67 and CD31 molecules with the efficacy of antitumor agents [40,47].The treatments with Rh_2_(H_2_cit)_4_ and Magh-Rh_2_(H_2_cit)_4_ showed a pronounced reduction of up to 50% in the number of Ki-67 positive cells (Figure 7E). Ki-67 is a nuclear protein linked to the cell cycle and a cell proliferation marker that has predictive and prognostic value in breast cancer patients [48], thus improving the understanding of the drug response. We suggest that Ki-67 reduction induced by Rh_2_(H_2_cit)_4_ and Magh-Rh_2_(H_2_cit)_4_ is associated with the tumor growth suppression observed in these treatments.

High levels of CD31 (angiogenesis marker) are correlated with poor prognosis and decreased survival of breast cancer patients [49]. Studies demonstrated that during prostate cancer treatment with cryoablation a positive correlation between necrosis and hypoxia and negative correlation with microvessel density was observed through the decrease in CD31 expression and the increase in necrosis area [50]. Further, another study demonstrated that tumor growth delay is associated with decrease in microvessel density and increase in tumor cell apoptosis [51]. Thus, the inhibition of tumor angiogenic factors is important since they are correlated with the metastasis process [52] and cell death [53].

Histopathological analysis showed numerous necrosis areas and low Ki-67 and CD31 expression in 4T1 tumor tissues treated with Rh_2_(H_2_cit)_4_ and Magh-Rh_2_(H_2_cit)_4_. Therefore, we suggest that the antitumor efficacy of these formulations is correlated with a reduction in Ki-67 and CD31 molecules, which indicates a decrease in both the cellular proliferation and microvessel network development. Overall, the histopathology, immunohistochemistry and regression tumor findings are consistent and linked.

## Conclusion

In summary, we demonstrated that both Rh_2_(H_2_cit)_4_ and Magh-Rh_2_(H_2_cit)_4_ formulations exhibited antitumor effects against 4T1 orthotopic breast cancer cell line following intratumoral administration. This antitumor effect was followed by inhibition of both cell proliferation and microvascularization and by tumor tissue injury characterized as necrosis and fibrosis. Remarkably, this is the first published report demonstrating the therapeutic efficacy of Magh-Rh_2_(H_2_cit)_4_. This treatment prolonged the survival period of treated mice without inducing apparent systemic toxicity, which strengthens the case for its use in future breast cancer therapeutic applications.

## Methods

### Preparation of the rhodium (II) citrate and rhodium (II) citrate-loaded maghemite nanoparticles

The maghemite nanoparticles (Magh-citrate) and rhodium citrate, Rh_2_(H_2_cit), as well as the rhodium(II) citrate-loaded superparamagnetic iron oxide nanoparticles (Magh-Rh_2_(H_2_cit)_4_), (Figure 1) were prepared and characterized as previously described [23]. Briefly, Rh_2_(H_2_cit)_4_ was synthesised by exchange trifluoroacetate ligands from the precursor rhodium(II) trifluoroacetate by citrate ligands. The compound was obtained as a green aqueous solution with a standardised concentration of 0.054 mol L^-1^. Maghemite nanoparticles were synthesised by alkaline co-precipitation of Fe^2+^ and Fe^3+^ ions. The particles obtained in the magnetite (Fe_3_O_4_) phase were oxidised to maghemite (γ-Fe_2_O_3_) by bubbling of oxygen gas and were subsequently purified by dialysis with deionised water for several days ([Fe] = 0.37 M). The Magh-Rh_2_(H_2_cit)_4_ was prepared using 5 mL of the colloidal dispersion with 1 mL of Rh_2_(H_2_cit)_4_ and stirred for 24 hours.

### Orthotopic tumor cell implantation and treatment

Balb/c female mice (12 weeks old) were purchased from Cemib-UNICAMP (São Paulo, Brazil). All mice were maintained in plastic cages under standard conditions of 12 h dark/light cycle. The mice, weighing 20-25 g, were fed with standard diet and water *ad libitum*. All experiments described were approved by the Animal Research Ethics Committee of the University of Brasilia - Institute of Biologic Sciences, Brazil.

The 4T1 breast carcinoma cells were thawed and cultivated in flasks with Dulbecco’s modified eagle’s medium (DMEM) supplemented with 1% penicillin and 10% fetal bovine serum (FBS) at 37°C in a humidified atmosphere 5% CO_2_. Two weeks later, Balb/c mice were anesthetized with ketamine (80 mg/kg) and xilazin (10 mg/kg) via intraperitoneal. Then, 2×10^4^ 4T1 cells (in suspension in 50 μL serum-free DMEM) were injected (1 mL-gauge needle) in their mammary gland, which is the natural primary microenvironment of breast tumor occurrence. Seven days after implantation of 4T1 cells, mice were divided into four groups (n=8/group), and each group was treated with 50 μL of (1) Rh_2_(H_2_cit)_4_, (2) Magh-Rh_2_(H_2_cit)_4_, (3) Magh-citrate or (4) water. Peritumoral injections were carried out every three days, totalizing seven applications of 0.3 mg/kg rhodium (II) citrate (total dose of Rh_2_(H_2_cit)_4_ was 2 mg/kg).

Mice treated with Magh-citrate received the same iron concentration and nanoparticle amount found in Magh-Rh_2_(H_2_cit)_4_ (0.37 M and 2.4×10^15^ particles). Animals without tumor and without treatment (healthy) were also included in this study as control groups. On the 23^rd^ experimental day after tumor implantation, the mice of each experimental group were euthanized and the tumor, liver, kidney, and lung were collected to perform antitumor and systemic toxicity analysis as described in items 2.4, 2.5 and 2.6.

### Systemic toxicity assessment in mice bearing 4T1 breast cancer

In order to evaluate potential systemic toxicity induced by the treatments, analyses of macroscopic aspects, histology, blood, DNA fragmentation and cell cycle were performed. Treated mice were continuously monitored for relevant indexes such as weight loss, diarrhea, skin ulcers and deaths.

Before euthanasia, which was by cervical dislocation, animals were anesthetized with the mixture of xylazine and ketamine described previously (item 2.3). Blood samples (1 mL/animal) collected by cardiac puncture were used to carry out hemogram and biochemical dosages of serum alanine aminotransferase (ALT), iron and creatinine. Hemogram was processed in a multiple automated hematology analyzer (XZ 2100 Sysmex equipment) and serum biochemical analyses were run on the automated chemistry analyzer ADVIA 2400 (Siemens), using the appropriate Advia chemistry reagents, protocols and controls.

After euthanasia, bone marrow (BM) cells were collected from femurs and resuspended in one milliliter of fetal bovine serum (FBS, Gibco) to perform DNA fragmentation and cell cycle analyses, which is a rapid detection method of chromosome damage and interference with cell mitosis caused by several agents [54]. Cells were fixed in cold 70% ethanol, and stored overnight at -30°C. The cells were centrifuged and incubated with 300 μL of lysis buffer (0.1% sodium citrate, 0.1% Triton X-100 and 20 μg/mL of propidium iodide, diluted in PBS pH 7.4) for 30 min at room temperature and protected from light. DNA fragmentation and the cell cycle were analyzed using FACS Calibur flow cytometry (Becton & Dickenson, USA) and a total of 10,000 events were collected per sample. Histopathology analysis of the liver, kidneys and lungs was also performed in order to verify possible toxic effects induced by treatments.

### Tumor regression and survival analysis

To evaluate tumor regression, tumors were surgically removed, their width and length measured by a digital pachymeter (Stainless, hardened), and their respective volumes calculated according to the formula of Yanase et al. (1998): length × width^2^ × 0.52 [55]. Animals that had died were submitted to necropsy, and the time of their death was recorded.

### Histopathology and immunohistochemistry analysis

Tumors were fixed in 10% phosphate-buffered formalin overnight (room temperature), transferred to 70% ethanol, included in paraffin using an automatic tissue processor (OMA® DM-40, São Paulo, Brazil), cut to 5 μm of thickness in a Leica RM2235 manual microtome (Leica Microsystems, Nussloch, Germany) and stained with hematoxilin-eosin (HE) or Perls Prussian Blue for histological analyses (light microscopy). Histological sections were examined to verify the presence of nanoparticles, cell proliferation pattern, pleomorphism, degree of cell differentiation and cell death.

Immunohistochemical analyses were performed in order to analyze cell proliferation by Ki-67 staining and vascularization by CD31 staining in the tissues. After paraffin removal and hydration, histological sections were immersed in citrate buffer (3 mM, pH 6.0) for 10 minutes at 120°C for antigen retrieval. Subsequently, non-specific binding sites were blocked with 3% normal serum or BSA. Afterward, the sections were incubated with anti-Ki-67 (1:200 *Abcam, ab15580*) or CD31 antibodies (1:200 Dako, K4067, Glostrup, Denmark) for 2 h at room temperature, washed and then incubated with biotinylated secondary antibodies for 20 min followed by avidin-biotin complex (LSAB-HRP Kit, Dako, K0690, Glostrup, Denmark). After washing, sections were incubated with diaminobenzidine substrate and counterstained with Mayer's hematoxylin.

All cells were counted in five consecutive microscopic high power fields (400x) using an integration graticule (CARL ZEISS-4740680000000-Netzmikrometer 12.5×). At this magnification, each field has an area equal to 0.015625 mm^2^; thus a total area equal to 0.078125 mm^2^ was analyzed in each specimen.

### Statistical analysis

To evaluate differences in tumor volume in each experimental group, after the treatments in Balb/c mice bearing 4T1 breast carcinoma, the following tests were run: inhibition of cell proliferation (by quantification of positive Ki67 staining cells number) in 4T1 breast carcinoma tissue; levels of serum ALT, creatinine and iron and peripheral blood counts (in order to verify the systemic effects of the treatments with Rh_2_(H_2_cit)_4_ and Magh-Rh_2_(H_2_cit)_4_); and in the proportion of DNA fragmentation or the cell cycle phase number of bone marrow of mice, the one-way Analysis of variance (ANOVA) was performed. When statistically significant differences were found, analysis was complemented by the Tukey test or the Bonferroni method. Before the intragroup comparison, the Shapiro Wilk test was conducted, to check whether each variable was normally distributed. Between-group comparisons of weight on different days after tumor transplantation were performed using a two-way ANOVA with post-hoc Dunnet test.

To describe the survival prolongation effect after the different treatments in Balb/c mice bearing 4T1 breast carcinoma, the Kaplan-Meier statistical method was used to generate survival curves. Then, these survival curves were compared using the Mantel-Haenszel log-rank test.

Data were presented as means ± SE. The significant level adopted was 5%. Calculations were done using the SPSS, Inc., Chicago, IL software (version 17.0). All plots were generated using GraphPad Prism 5.0 (GraphPad Software, La Jolla, Calif.).

## Competing interests

The authors declare that they have no competing interests.

## Authors’ contributions

MLBC was the principal investigator and takes primary responsibility for the paper. MLBC, ZGML and SNB participated in the design of the study and SNB co-ordinated the research. MLBC, RCAP, RGSO and LAMT performed the laboratory work for this study. GAJ carried out the genotoxic test and LSV participated in the histopathological analysis. ALB carried out the immunoassays and ALMV helped to draft the manuscript and conduct data analysis. ARS synthesized the rhodium (II) citrate and rhodium (II) citrate-loaded nanoparticles, ICRS was responsible for statistical analysis; MLBC, GAJ and ALMV wrote the manuscript. All authors read and approved the final manuscript.

## Authors’ information

Marcella Lemos Brettas Carneiro is the first author of this work.
